# The accuracy of a three-dimensional face model reconstructing method based on conventional clinical two-dimensional photos

**DOI:** 10.1186/s12903-022-02439-0

**Published:** 2022-09-19

**Authors:** Bochun Mao, Jing Li, Yajing Tian, Yanheng Zhou

**Affiliations:** Department of Orthodontics, Peking University School of Stomatology & National Center of Stomatology & National Clinical Research Center for Oral Diseases & National Engineering Research Center of Oral Biomaterials and Digital Medical Devices, Beijing Key Laboratory of Digital Stomatology, 22 Zhongguancun South Avenue, Haidian District, Beijing, 100081 People’s Republic of China

**Keywords:** Three-dimensional diagnosis and treatment planning, Digital models, Facial reconstruction, Face scan

## Abstract

**Background:**

This study aims to investigate the accuracy of a three-dimensional (3D) face reconstruction method based on conventional clinical two-dimensional (2D) photos.

**Methods:**

Twenty-three patients were included, and Character Creator v3.2 software with the Headshot v1.0 plugin was used for 3D face model reconstruction. Various facial landmarks were finely adjusted manually to refine the models. After preprocessing and repositioning, 3D deviation analysis was performed. The accuracy of the landmarks in different dimensions was determined, and twelve facial soft tissue measurements were compared to validate the clinical potential of the method.

**Result:**

The reconstructed 3D face models showed good facial morphology with fine texture. The average root mean square errors between face scan models and reconstructed models at perioral area (1.26 ± 0.24 mm, 95%CI: 1.15–1.37 mm) were significantly smaller than the entire facial area (1.77 ± 0.23 mm, 95%CI:1.67–1.88 mm), *P* < 0.01. The deviation of menton of soft tissue was significantly larger than pronasale (*P* < 0.01). The deviations of all landmarks in the Y-direction were significantly larger than those in the other 2 dimensions (Y > Z > X, *P* < 0.01). A significant difference (*P* < 0.05) of approximately 1.5 mm was found for facial height. Significant differences (*P* < 0.05) were also identified in the remaining 6 soft tissue measurements, with average deviations no greater than 0.5 mm (linear measurement) or 1.2° (angular measurements).

**Conclusion:**

A 3D face modeling method based on 2D face photos was revealed and validated. The reconstruction accuracy of this method is clinically acceptable for orthodontic measurement purposes, but narrow clinical indications and labor-intensive operations remain problems.

## Background

The concept of orthodontic aesthetics has evolved from the “full complement of teeth”, which was proposed by Edward H. Angle in the early 1900s [1], to the emphasis on facial soft tissue aesthetics. An increasing number of chief complaints of patients who pursue orthodontic treatment now involve profile change requirements. Therefore, it has become routine to include facial measurements in orthodontic treatment plans.

Two-dimensional (2D) facial measurement strategies have been developed based on 2D facial records, which can aid in the diagnosis of soft tissue aesthetic problems to some extent. However, due to the restriction of the dimension of the 2D measurement, drawbacks such as inaccuracy, the inaccessibility of three-dimensional (3D) facial morphology, and problematic superimposition of the anatomical structures have been identified in a few studies [2, 3]. These major drawbacks limit the development of 2D measurements in the field of orthodontics. It has also been 20 years since 3D imaging systems were initially introduced to dental clinical practice; these systems are promising and can provide highly accurate, detailed diagnoses of facial soft tissue for orthodontic and maxillofacial treatment planning [4, 5]. 3D Facial profile is considered dependent on dentoskeletal tissue and overlying soft tissue. Studies indicated the strong correlation between the 3D soft tissue facial profile and the skeletal pattern [6, 7]. For example, for a typical class II hyperdivergent patient, sagittal and vertical inharmonious statuses cause retrusive and clockwise rotated mandible that leads to convex facial profile with excessive lower facial height. In contrary, typical class III hypodivergent pattern always represents as concave profile with counterclockwise rotated mandible.

There are several feasible ways to obtain 3D face models, including cone-beam computed tomography (CBCT), magnetic resonance imaging (MRI), and optical face scan. Face scans have been the gold standard for facial modeling due to their high accuracy (0.2 mm), leading to common usage and growing popularity among plastic surgeons, orthodontists, and maxillofacial surgeons [5]. In recent years, several portable and affordable 3D scanners have been made available to orthodontists, which further strength the 3D facial analysis [8].

However, the rapid development of orthodontic 3D facial analysis requires more 3D face data, especially for paired facial models before and after orthodontic treatment. Due to the essence of orthodontic treatment, approximately 2–3 years are required to finish a treatment, limiting the currently available 3D face models for relevant studies. 2D facial photo documentation has always been important clinical data during orthodontic treatment. A 3D face model reconstruction method based on 2D photos can markedly enhance the available data for 3D facial analysis, which can make past cases ‘alive’ for 3D facial analysis. We believe with this method, orthodontic 3D facial measurement can be further developed.

Currently, the existing methods of reconstructing 3D face models based on 2D photos are limited to the field of computer science without medical consideration [9, 10]. These methods primarily focus on the robustness of reconstruction of the entire face area instead of the accuracy of the models, especially for the perioral area [11]. Additionally, the accuracy of those methods was not clear in clinical settings.

Therefore, this study aims to investigate the accuracy of a 3D face modeling method based on 2D face photos. After a literature review and preliminary experiments, which included the exploration of software including Meshroom (Alicevision Association) and Bellus3d (Campbell, CA, USA), we chose the 3D animation modeling software Character Creator v3.2 with the Headshot v1.0 plugin (Reallusion Inc) for this study.

## Methods

### Study sample

This study was approved by the Institutional Review Board of Peking University School and Hospital of Stomatology (No. PKUSSIRB-202058135). The sample size was calculated to detect a significant mean difference of 0.42 with a standard deviation of 0.68 based on a pilot study. The significance level was set at 0.05, and the power of the test was set as 0.80. Accordingly, it was determined that a minimum number of 23 patients were required for this paired design study. Thus, 23 patients (13–29 years old, mean ± SD: 20.70 ± 5.36 years), 14 females and 9 males, ready for pre-orthodontic examinations from January 2021 to April 2021 at Department of Orthodontics, Peking University School of Stomatology were enrolled in the study after providing written informed consent. The exclusion criteria were as follows: (1) subjects with a history of maxillofacial trauma or maxillofacial surgery; (2) subjects with obvious facial asymmetry or scars around the face; (3) patients with beard or moustache; and (4) subjects with face muscle spasm symptoms.

### 2D and 3D photo acquisition process

**Fig. 1:**
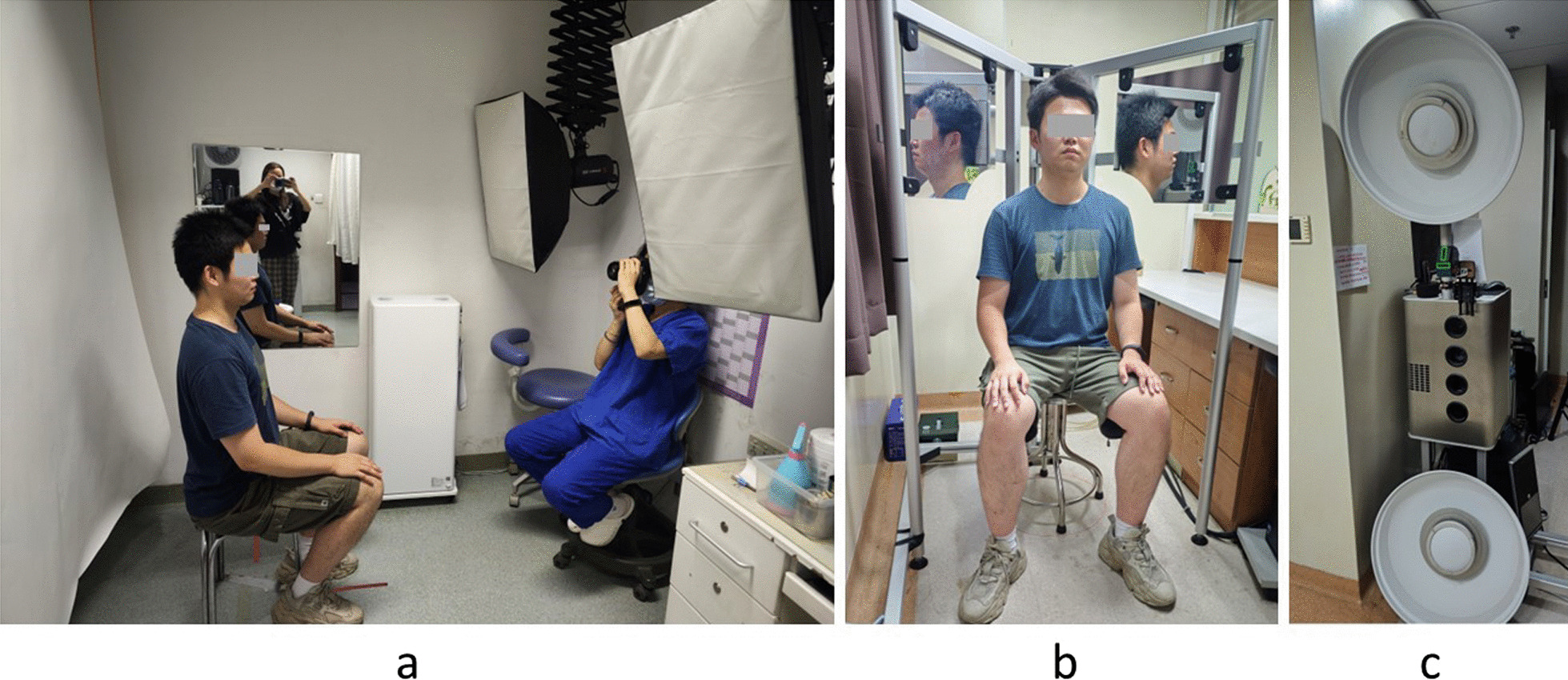
**a** A patient positioned for standardized 2D photo-taking; **b** A patient positioned for standardized 3D facial scanning; **c** The photographic equipment of the 3D facial scan system

For 2D photo acquisition, a Canon EOS 60D camera with a 60-mm prime lens was used. Photos were taken according to the standard of the American Board of Orthodontics (ABO) inside a room with normal lighting conditions and fluorescent lights (Fig. 1a). The camera was set up to a shutter speed of 1/125 with an aperture of F7.1 and ISO at 100. The filming standards for 2D face photos are as follows: (1) the camera was placed 150 cm away from the subject’s face approximately at the same height as the midface; (2) the subjects were asked to wear nether make-up, glasses or scarf that might cover the facial area, and the forehead and ears were completely exposed; (3) the subjects were asked to look straight forward with the head in the natural head position with their teeth in rest occlusion and their eyes open; (4) subjects were asked to relax their lips and perioral muscles in a natural head position; (5) subjects were asked to keep their interpupillary line horizontal to the viewfinder frame of the camera, which has horizontal and vertical trisection lines for reference; (6) the approximate center of viewfinder frame was the tip of the nose; and (7) after the front view photos were taken, subjects were asked to turn sideways slowly at 90° while keeping the head pitch angle, and the approximate center of viewfinder frame was 1.0 cm anterior to the tragus. Red marks were painted on the floor to ensure that the photo pairs were orthogonal.

3D face models were acquired with the 3D optical FaceSCAN3D system (3D-Shape, Erlangen, Germany) according to the filming standard [12] (Fig. 1b, c). The subjects were properly positioned, 1 m from the camera placed sideways and 90 cm from the scanner placed frontally. The filming standards for 3D face models were the same as the 2nd, 3rd, and 4th standards of 2D photo taking. The acquired 3D models were saved as object files (.obj) for further processing.

### Face model reconstruction process

**Fig. 2:**
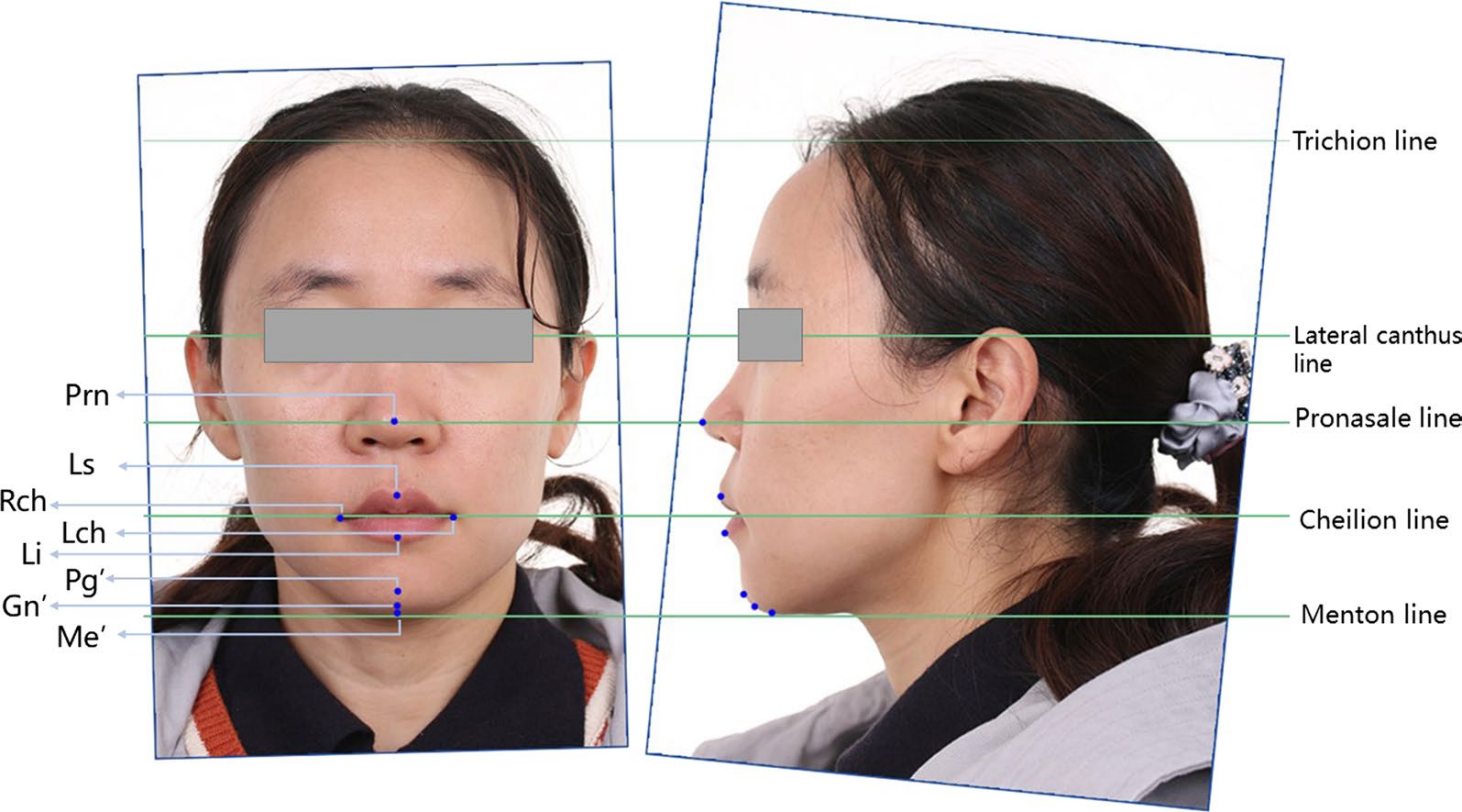
Reposition of 2D face photos. Front view photographs and left side view photographs were imported into Photoshop CS6 for scaling and alignment based on facial landmarks (Prn: pronasale, Ls: labrale superius, Li: labrale inferius, Lch: left cheilion, Rch: right cheilion, Pg’: pogonion of soft tissue, Gn’: gnathion of soft tissue, Me’: menton of soft tissue)

The repositioning of 2D face photos was first performed. As Fig. 2 shows, both front- and lateral-view photos were imported into Adobe Photoshop CS6 (Adobe Systems Inc., San Jose, United States). First, the front photos were rotated to ensure that the lateral canthus line was horizontal. Then, the vertical distance from the lateral canthus to the cheilion (H1) was measured. For the lateral view photos, the rotation center was pinned to the lateral canthus, and the picture was rotated to ensure the same vertical distance from the lateral canthus to cheilion as H1. Reference lines marking the trichion, the lateral canthus, pronasale, cheilion, and menton were used for repositioning.

**Fig. 3:**
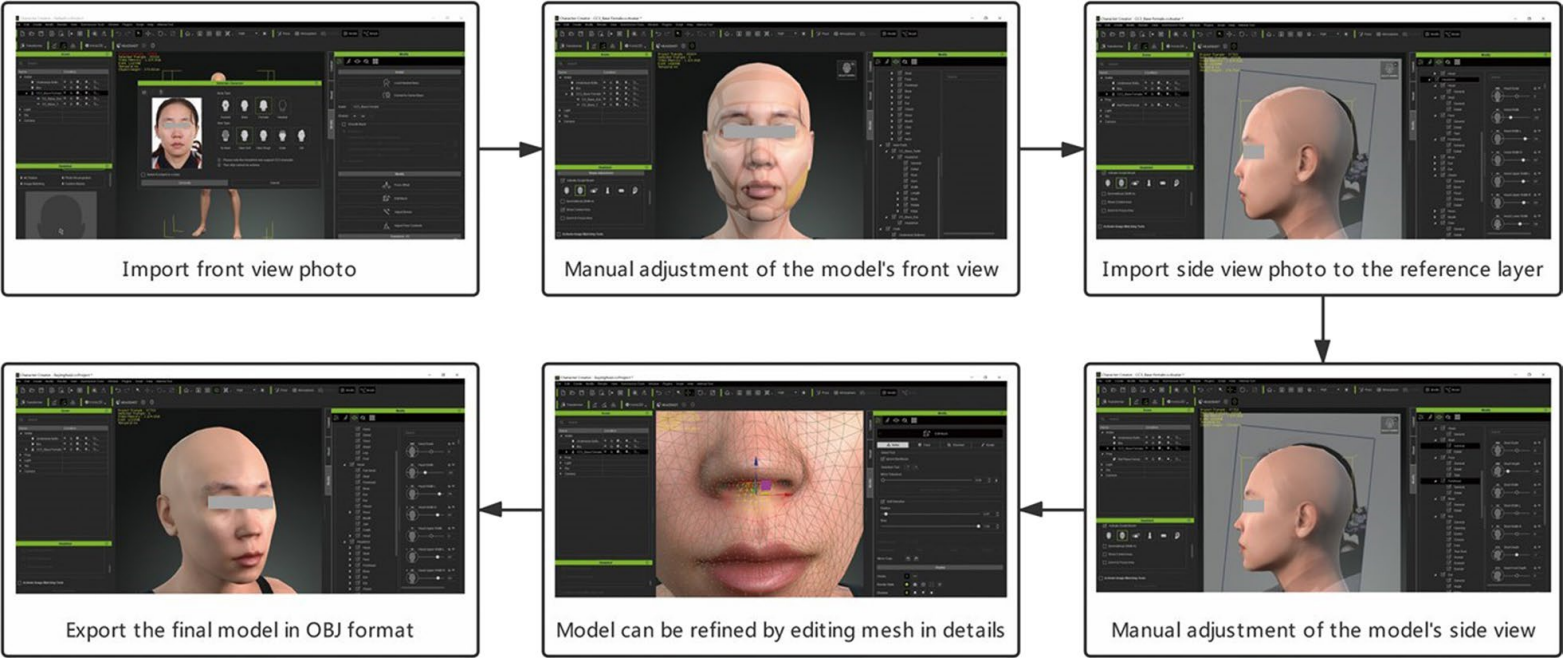
Workflow of face model reconstruction in Character Creator v3.2 (with Headshot v1.0 plugin)

Character Creator v3.2 with the Headshot v1.0 plugin was used for 3D face model reconstruction (Fig. 3). The repositioned front view photo was first imported into the Headshot v1.0 plugin in Pro mode. The primary features of the Headshot plugin include intelligent texture blending and head mesh creation. The neural rendering (NR) technique was used for face reconstruction. NR is based on machine learning (ML) techniques that are used to infer human face features from large amounts of training data. After the automatic modeling process, the focal lens was determined with the slider ‘Camera Settings’ to correct the lens distortion, and the ‘Activate Image Matching Tools’ was selected. Manual fine adjustment was then performed by comparing the superimposed front view photo with the model in detail. ‘Re-Project Photo’ was then selected to optimize the facial texture after the front view of the model was refined. Then, the lateral view of the model was adjusted. The standard reference side view was determined by clicking ‘Content; Camera; Headshot Ref; Ref Camera’ in sequence. The lateral view photo was imported into a standard reference layer by clicking ‘Content; Prop; Headshot Ref; Ref Plan base; Import photo’ in sequence. The reference layer was translated vertically to match the height of the model. Because the height and width of all anatomical structures were determined during the front view adjustment, only the depth (sagittal plane) of anatomical structures required adjustment. When adjusting morph sliders, front facing planar morphs will not affect the depth value of the side facing morphs, and vice versa.

Despite all the adjusted panels provided by the plugin, the model could also be adjusted in detail by ‘Editing Mesh’. After the reconstruction of the model was done, ‘Re-Project Photo’ was again selected to optimize the facial texture before it was exported in OBJ format.

### 3D-deviation analysis

In Geomagic Control (Geomagic, Morrisville, North Carolina, USA), models were segmented to preserve only facial area. Construction and unification of the 3D coordinate system was performed according to the method revealed in a previous study [12]: A 3D coordinate system was generated with the midpoint of the bilateral tragion as the origin, and the X axis was set as the line through bilateral tragion, and soft tissue Frankfort horizontal (FH) plane, determined by both tragion points and the right suborbital point, was set as the X–Y plane. Within this coordinate system, the coronal, sagittal, and axial reference planes were set as the x-, y-, and z-axis, respectively. The left, superior, and anterior directions were considered positive for the respective axes.

**Fig. 4:**
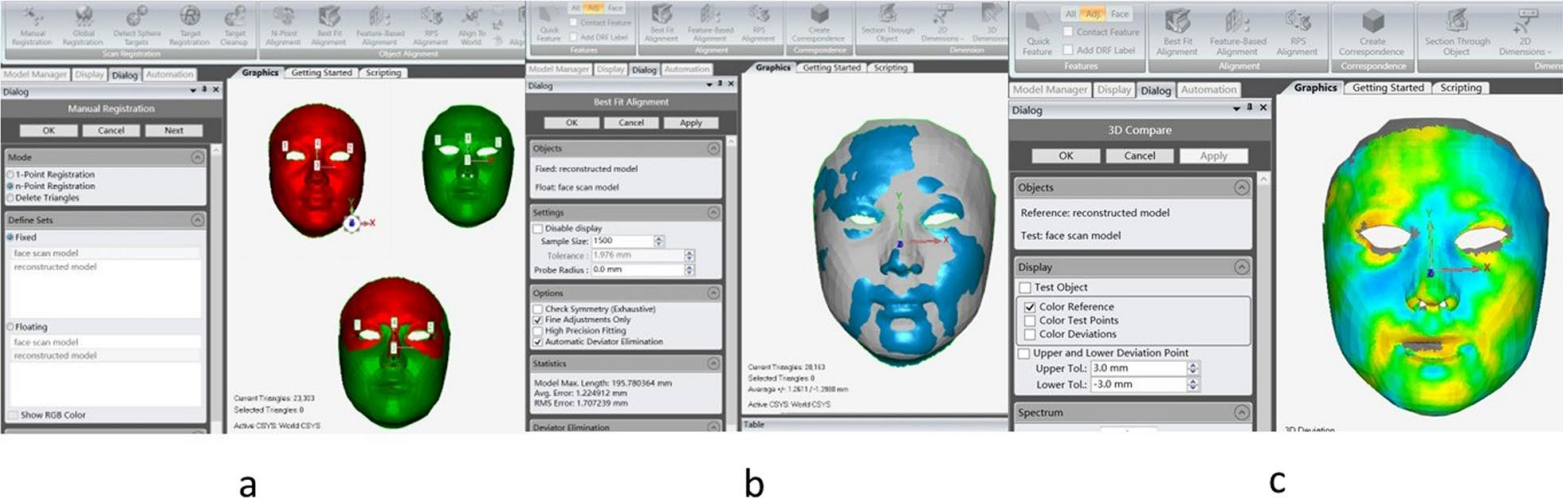
The two-step registration strategy used in this study. **a** manual registration was carried out with 4 manually set landmarks (bilateral canthus, nose tip, and soft tissue nasion); **b** best fit alignment was carried out for the whole face model; **c** the 3D deviation analysis

Different from the conventional registration strategy [13] for the 3D face model, which uses the forehead, upper nasal dorsum, and zygoma for surface registration, a two-step registration strategy was chosen in this study for the registration of 3D scan facial models and 3D face models reconstructed from 2D images. Firstly, manual registration was carried out with 4 manually set landmarks (bilateral canthus, nose tip, and soft tissue nasion, Fig. 4a). Then best fit alignment was carried out for the whole face model (with at least 50 iterations, precision of the registration to at least 0.1 mm, the polygon surface registration percentages to the maximum 100% and range of tolerance of 2 mm [14], Fig. 4b). This registration strategy was chosen because the reconstructed model was determined mainly on the adjustment of facial landmarks, such as bilateral canthus, nose tip, and soft tissue nasion, and the deviation of these landmarks should be at a low level compared with plain surfaces such as the forehead.

Eight landmarks (Prn: pronasale, Ls: labrale superius, Li: labrale inferius, Lch: left cheilion, Rch: right cheilion, Pg’: pogonion of soft tissue, Gn’: gnathion of soft tissue, Me’: menton of soft tissue) were identified on each model to investigate the accuracy of the reconstructed models (Fig. 2) [15]. 3D deviation analysis was carried out (Fig. 4c). The values of the 3D deviations were expressed using root mean square root mean square error (RMSE), which was calculated using the following formula:

$${\text{RMS}} = \frac{1}{\sqrt n }\sqrt {\mathop \sum \limits_{i = 1}^{n} \left( {x_{1,i} - x_{2,i} } \right)^{2} }$$

where x_1_ is to the measurement point on reference Model i, x_2_ is to the measurement point on test Model i, and n is the total number of measurements for each specimen.

The differences were represented along a color spectrum with values associated with each color. The absolute error and deviations of each landmark were recorded in 3 dimensions. Two independent operators (B. M, Y. T), who were blinded to each other’s operation, participated in the landmark setting, and the operations were repeated 1 week after the first time. According to previous studies, the clinical acceptable accuracy of 3D facial model was set to 2 mm [16, 17].

### Soft tissue measurement

**Fig. 5:**
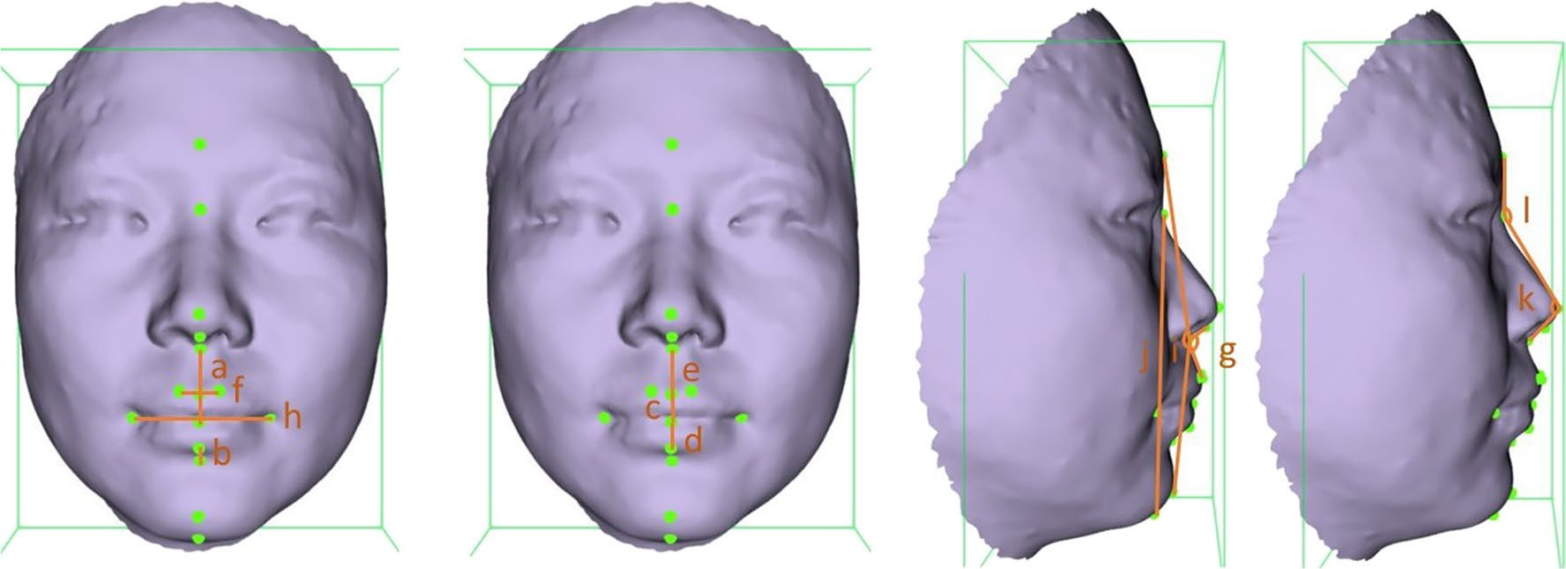
The Linear and angular soft tissue measurements used in this study. **a** upper lip height; **b** lower lip height; **c** upper vermilion height; **d** lower vermilion height; **e** philtral length; **f** philtral width; **g** nasolabial angle; **h** labial fissure width; **I** facial convexity; **j** facial height; **k** nasal angle; **l** nasofrontal angle

Twelve commonly used 3D facial soft tissue linear or angular measurements were chosen based on previous studies (Fig. 5) [7, 15]. The definitions of the soft tissue measurements are shown in Table 1. Similar to the landmark setting, 2 independent operators (B. M, Y. T), who were blinded to each other’s measurements, participated in the measurements, and the measurements were repeated 1 week after the first time.

**Table1:** Definition of the soft tissue measurements (Sn: Subnasale; Sto: Stomion; Sl: Sublabial; Prn: Pronasale; Ls: Labrale superius; Li: Labrale inferius; (L,R) ch: (Left, Right) cheilion; (L,R) Cph: (Left, Right) Crista Philtri; Gl: Glabella; N’: Nasion of soft tissue; Pg’: Pogonion of soft tissue; Gn’: Gnathion of soft tissue)

Measurement	Definition
Upper Lip Height (Sn-Sto)	Height of the entire upper lip measured from subnasale to stomion superius
Lower Lip Height (Sl-Li)	Vertical measurement of the lower lip below the vermilion
Upper Vermilion Height (Ls-Sto)	Height of the upper lip vermilion measured from labrale superius to stomion superius
Lower Vermilion Height (Li-Sto)	Height of the vermilion segment of the lower lip
Philtral Length (Sn-Ls)	Distance between the nasal bone/base and midline upper lip vermilion border
Philtral Width (CphR-CphL)	Distance between the philtral ridges, measured just above the vermilion border
Nasolabial Angle (Prn-Sn-Ls)	Angle at subnasale subtended by side columella—labrale superius
Labial Fissure Width (Lch-Rch)	Distance between the commissures of the mouth
Facial Convexity (Gl-Sn-Pg’)	Angle at subnasale subtended by side glabella—pogonion
Facial Height (N’-Gn’)	Vertical height (length) of the face or viscerocranium
Nasal Angle (N’-Prn-Sn)	Angle at pronasale subtended by side nasion – subnasale
Nasofrontal Angle (Gl-N’-Prn)	Angle at nasion subtended by side glabella—pronasale

### Statistical analysis

All data were analyzed with SPSS 19.0 (SPSS, USA). The accuracy of model reconstruction was assessed with the independent-samples t test, and the soft tissue measurements were assessed with the paired t test after normal distribution of the data was determined. One-way ANOVA with the SNK test was used to evaluate the deviation of landmarks after the normal distribution of the data was determined. Kruskal–Wallis tests were used to evaluate the accuracy of the landmarks in different dimensions when variance was uneven. Differences with *P* < 0.05 were considered statistically significant.

### Method error

The intrarater and interrater reliability of measurements were assessed using 2-way absolute agreement intraclass correlation coefficient (ICC) on a mean of measurements. The Bland–Altman analysis was used to assess the difference between the measurements obtained by the 2 methods.

## Result

**Fig. 6:**
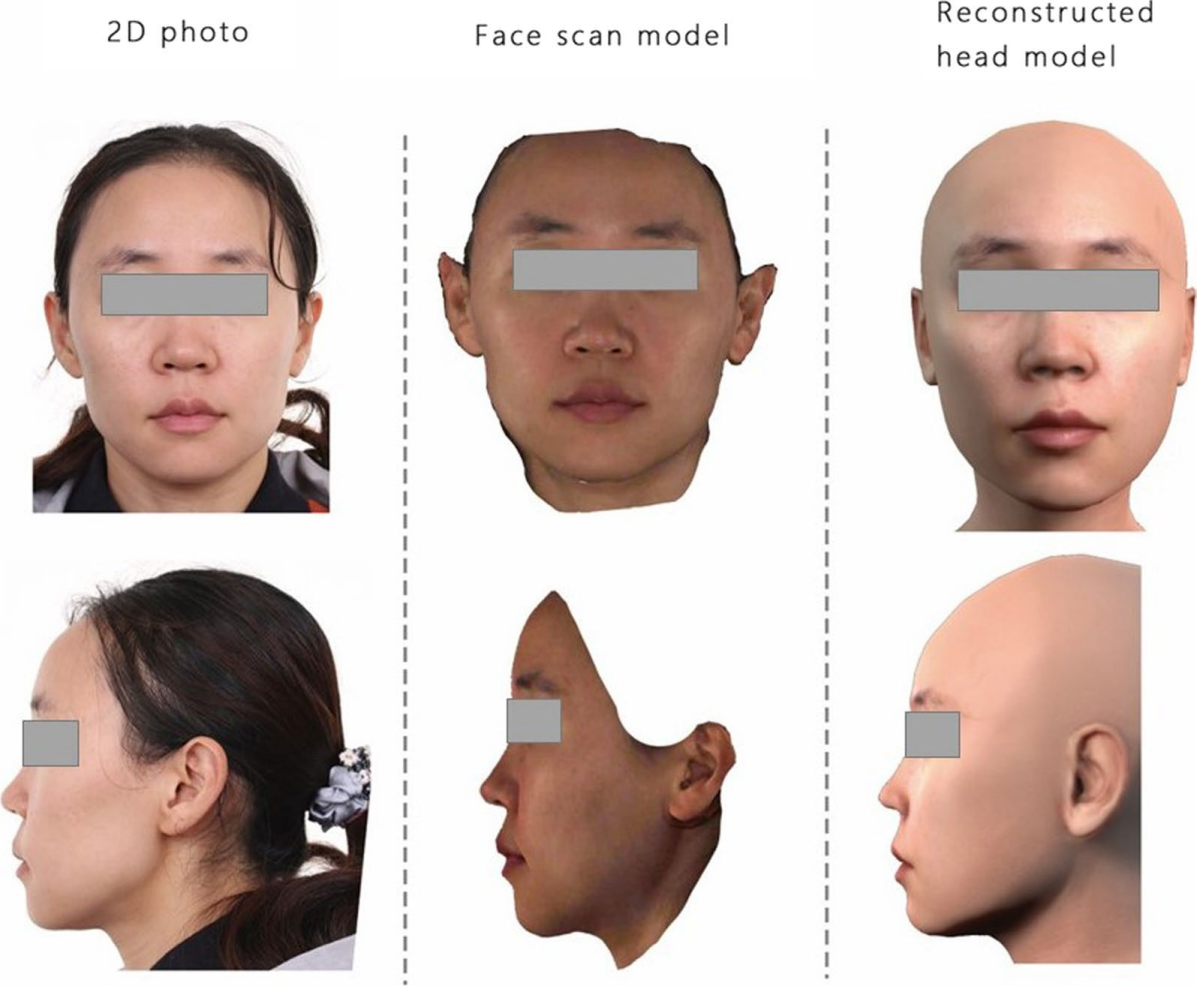
2D face photo, face scan model, and reconstruction model of a patient

3D face models were successfully reconstructed from 2D face photos with the proposed method. Figure 6 shows 1 subject out of the 23 patients. The reconstructed 3D face models showed good facial morphology with fine texture, demonstrating that high-resolution 2D images were successfully mapped to the meshes to create photorealistic representations of the subjects’ faces. The perioral area of a reconstructed model was finely built in both morphology and texture, which was also supported by the numerical results.

**Fig. 7:**
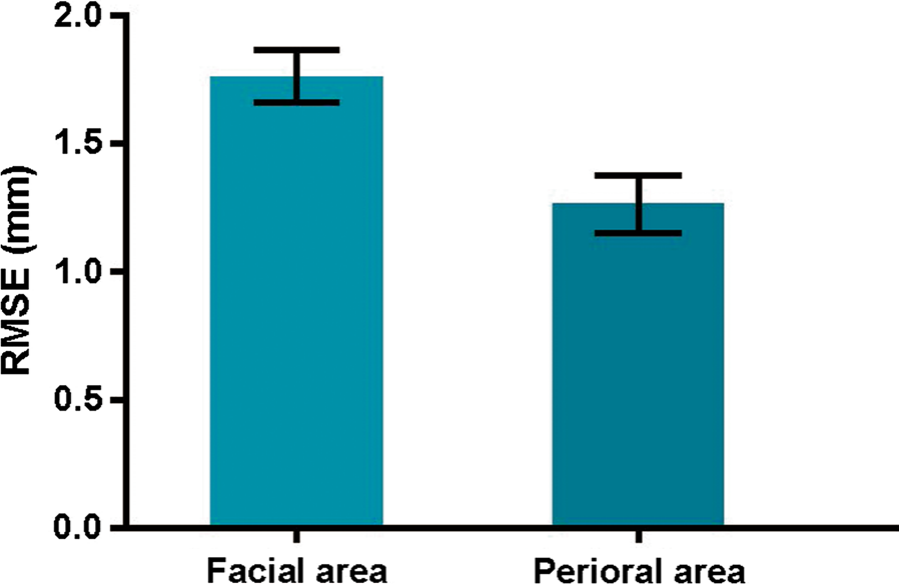
Root mean square error (RMSE) of the 3D-deviation analysis of facial area and perioral area (Mean ± 95%CI, mm)

**Fig. 8:**
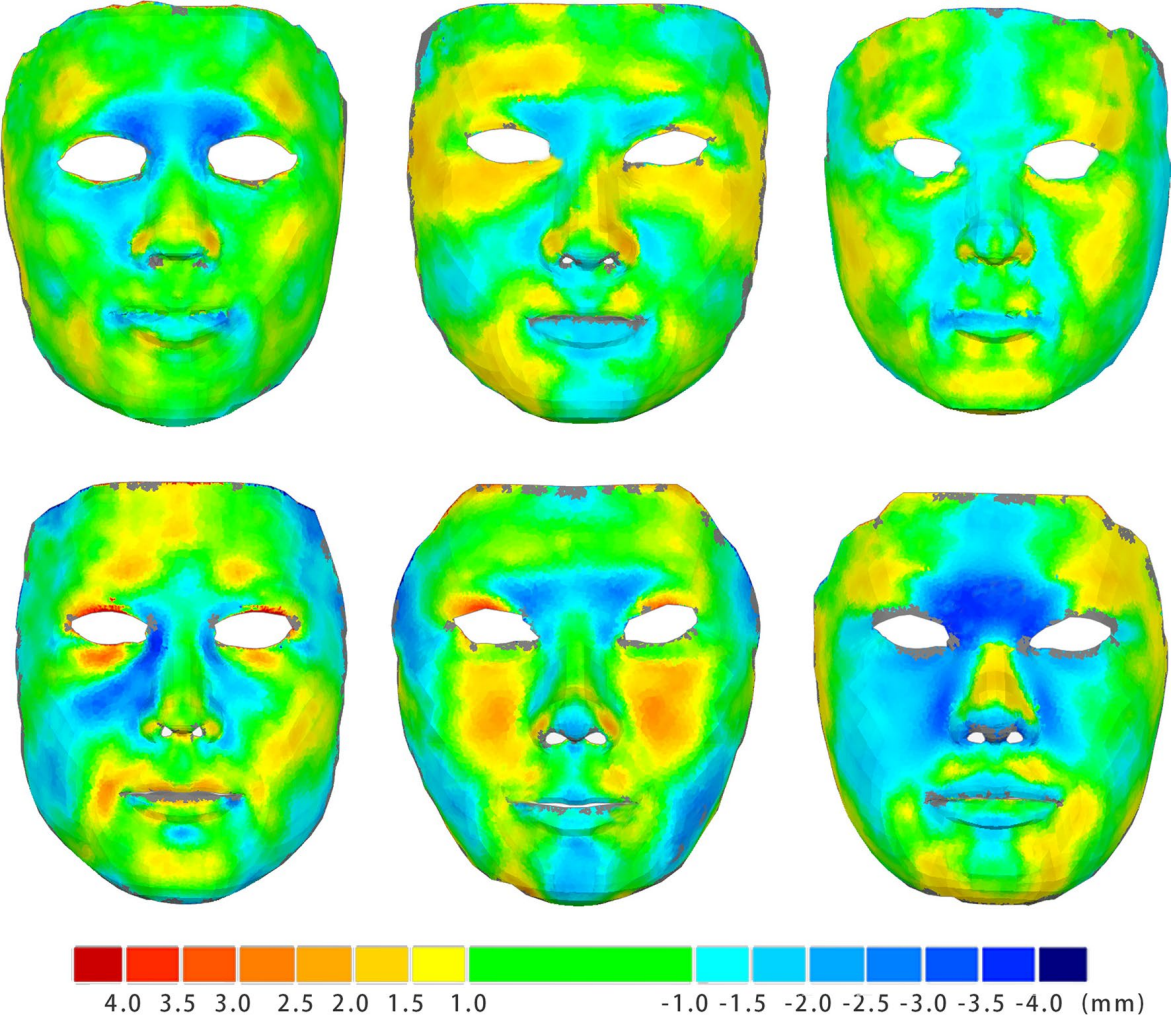
Cloud maps of 3D deviation between face scan models and reconstruction models of 6 patients

As shown in Fig. 7, the average RMSEs between face scan models and reconstructed models at perioral area (1.26 ± 0.24 mm (Mean ± SD), 95%CI: 1.15–1.37 mm) were significantly smaller than the entire facial area (1.77 ± 0.23 mm (Mean ± SD), 95%CI:1.67–1.88 mm), *P* < 0.01. However, both 95% CIs of the deviations were smaller than 2 mm, which demonstrated that the models were clinically acceptable [16, 17]. As shown in the color-coded maps (Fig. 8), the forehead (anteriorly) and cheek (posteriorly) areas remained the most inaccurate, with RMSEs less than 3 mm.

**Fig. 9:**
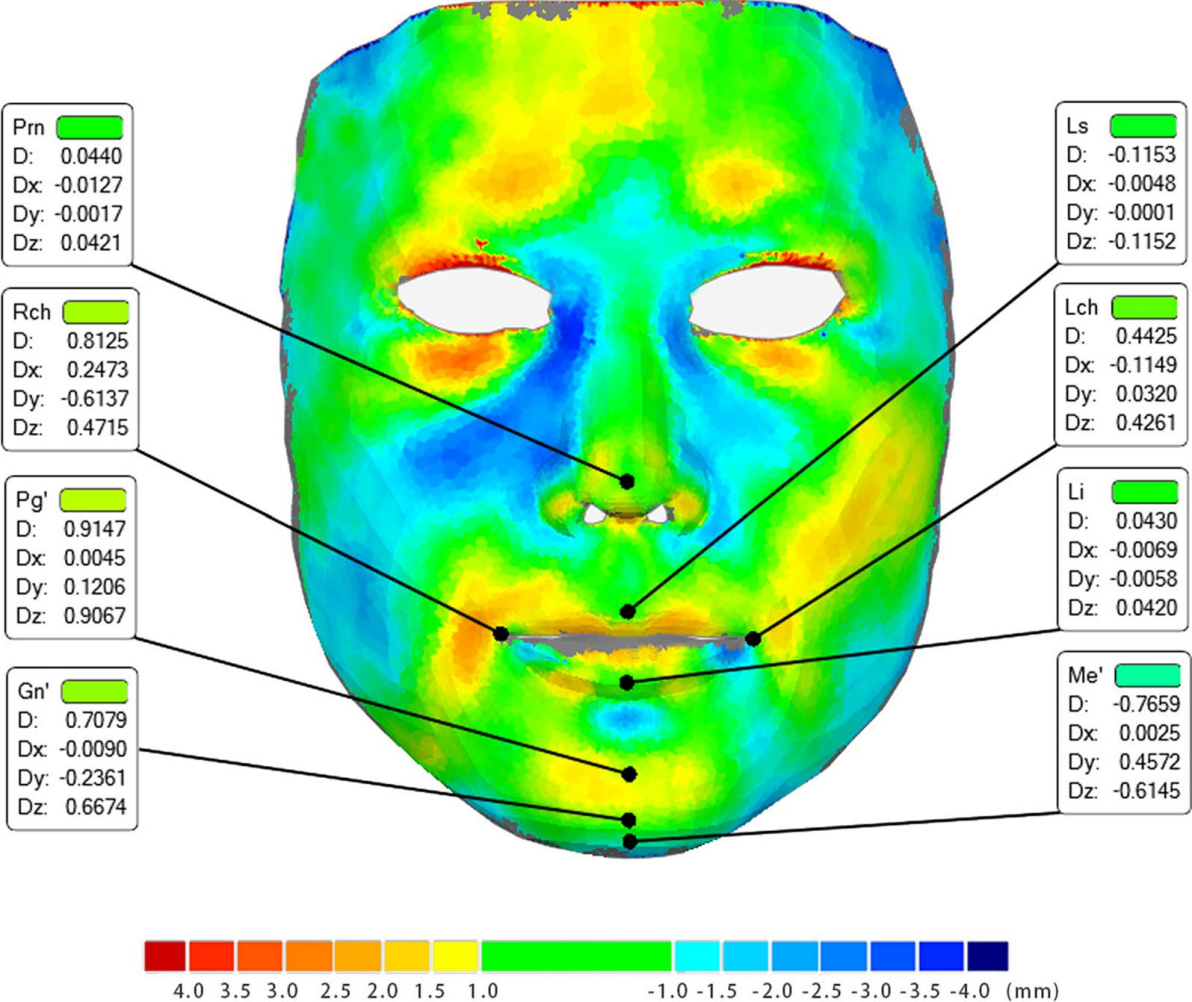
Color-coded map of 3D-deviation analysis of a typical subject. The deviation of all the landmarks is shown and denoted as follows: Prn = pronasale, Ls = labrale superius, Li = labrale inferius, Lch = left cheilion, Rch = right cheilion, Pg’ = pogonion of soft tissue, Gn’ = gnathion of soft tissue, and Me’ = menton of soft tissue

**Fig. 10:**
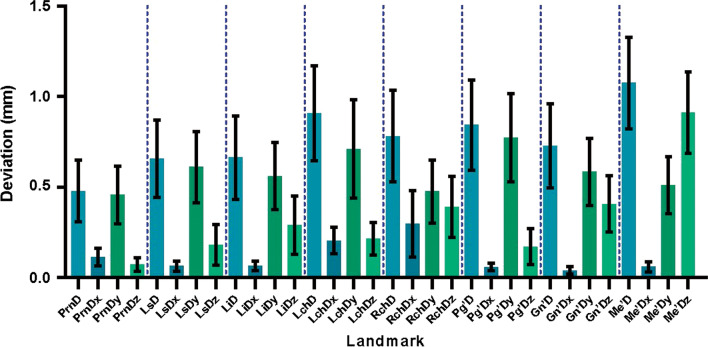
Deviations of landmarks in different directions (mean ± 95%CI, mm; LL: lower limit; UL: upper limit; D: total deviation, Dx: deviation in horizontal level, Dy: deviation in sagittal level, Dz: deviation in vertical level, Prn: pronasale, Ls: labrale superius, Li: labrale inferius, Lch: left cheilion, Rch: right cheilion, Pg’: pogonion of soft tissue, Gn’: gnathion of soft tissue, Me’: menton of soft tissue)

For the landmarks (Table 2, Figs. 9 and 10), there were no significant differences (*P* > 0.05) among the absolute mean errors between corresponding landmarks except for soft tissue (Me’) and pronasale (Prn). The deviation of the menton of soft tissue (Me’) was significantly larger than that of pronasale (Prn) (*P* < 0.01). Additionally, the deviation of all landmarks in different directions varied significantly. (Y > Z > X, *P* < 0,01). The mean absolute errors in the Y-direction were significantly greater than those in the other two directions. The deviations of all landmarks in all directions were within 1.5 mm, which was considered clinically acceptable.

**Table 2:** Deviations of landmarks in different directions (mm; D: total deviation, Dx: deviation in horizontal level, Dy: deviation in sagittal level, Dz: deviation in vertical level, Prn: pronasale, Ls: labrale superius, Li: labrale inferius, Lch: left cheilion, Rch: right cheilion, Pg’: pogonion of soft tissue, Gn’: gnathion of soft tissue, Me’: menton of soft tissue)

Landmark (mm)	D				Dx				Dy				Dz			
	Mean	SD	95%Ci (LL)	95%Ci (UL)	Mean	SD	95%Ci (LL)	95%Ci (UL)	Mean	SD	95%Ci (LL)	95%Ci (UL)	Mean	SD	95%Ci (LL)	95%Ci (UL)
Prn	0.48	0.39	0.31	0.65	0.11	0.11	0.07	0.16	0.46	0.37	0.3	0.62	0.07	0.09	0.04	0.11
Ls	0.66	0.49	0.44	0.87	0.06	0.06	0.04	0.09	0.61	0.45	0.41	0.81	0.18	0.26	0.07	0.29
Li	0.66	0.53	0.89	0.43	0.07	0.06	0.04	0.09	0.56	0.43	0.37	0.75	0.29	0.37	0.13	0.45
Lch	0.91	0.6	1.17	0.65	0.2	0.17	0.13	0.28	0.71	0.63	0.44	0.98	0.22	0.04	0.13	0.31
Rch	0.78	0.58	1.03	0.53	0.3	0.42	0.12	0.48	0.48	0.4	0.3	0.65	0.39	0.39	0.22	0.56
Pg’	0.84	0.57	1.09	0.59	0.06	0.05	0.04	0.08	0.77	0.56	0.53	1.02	0.17	0.23	0.07	0.27
Gn’	0.73	0.54	0.96	0.49	0.04	0.05	0.02	0.06	0.58	0.42	0.4	0.77	0.41	0.36	0.25	0.56
Me’	1.07	0.59	1.33	0.82	0.06	0.07	0.03	0.09	0.51	0.36	0.35	0.67	0.91	0.52	0.69	1.14

A comparison of 3D facial measurements made on soft tissues according to groups is shown in Table 3. No significant differences were identified in 5 measurements. However, a significant difference (*P* < 0.05) of approximately 1.5 mm was found in facial height (Nasion of soft tissue—Gnathion of soft tissue). Significant differences (*P* < 0.05) were also identified in the remaining 6 measurements, with average deviations no greater than 0.5 mm (linear measurement) or 1.2° (angular measurements).

**Table 3:** Comparison of 3D facial measurements made on soft tissues according to groups (SD: standard deviation; NS: not significant; *Significance level at *P* < 0.05; (Sn: Subnasale; Sto: Stomion; Sl: Sublabial; Prn: Pronasale; Ls: Labrale superius; Li: Labrale inferius; (L,R) ch: (Left, Right) cheilion; (L,R) Cph: (Left, Right) Crista Philtri; Gl: Glabella; N’: Nasion of soft tissue; Pg’: Pogonion of soft tissue; Gn’: Gnathion of soft tissue)

Measurement	Face scan group (mean ± SD)	Reconstruction group (mean ± SD)	Deviation (face scan Group-reconstruction group, mean ± SD)	P*
Upper lip height (Sn-Sto)	21.79 ± 1.12	21.34 ± 1.17	0.44 ± 0.71	< 0.01
Lower lip height (Sl-Li)	7.77 ± 0.56	8.29 ± 1.04	− 0.52 ± 0.85	< 0.01
Upper vermilion height (Ls-Sto)	8.98 ± 0.71	8.73 ± 0.64	0.25 ± 0.58	NS
Lower vermilion height (Li-Sto)	8.64 ± 0.79	8.78 ± 1.12	− 0.14 ± 0.75	NS
Philtral length (Sn-Ls)	14.60 ± 0.97	14.17 ± 1.14	0.43 ± 0.75	0.011
Philtral width (CphR-CphL)	12.90 ± 0.48	12.40 ± 0.77	0.50 ± 0.77	< 0.01
Nasolabial angle (Prn-Sn-Ls)	105.62 ± 7.59	106.70 ± 7.15	− 1.09 ± 2.80	NS
Labial fissure Width (Lch-Rch)	45.48 ± 2.24	44.86 ± 2.52	0.63 ± 1.77	NS
Facial convexity (Gl-Sn-Pg’)	167.07 ± 3.89	166.67 ± 3.97	0.40 ± 1.68	NS
Facial height (N’-Gn’)	115.72 ± 3.23	114.16 ± 3.18	1.56 ± 1.28	< 0.01
Nasal angle (N’-Prn-Sn)	118.35 ± 4.10	117.34 ± 4.61	1.01 ± 1.63	< 0.01
Nasofrontal angle (Gl-N’-Prn)	142.61 ± 4.47	143.73 ± 3.98	− 1.12 ± 1.93	0.011

The results from the ICC and Bland–Altman analysis are summarized in Table 4. The ICC for the intrarater reliability was above 0.85 for both investigators for all measurements, and ICC for the interrater reliability was above 0.82. These findings indicated that this method of measurement was quite stable and reliable. The Bland–Altman results indicated the 95% limits of agreement were all within 1.4 mm for linear measurements (except for facial height) and 2.5° for angular measurements.

**Table 4:** Summary of results of intraclass correlation coefficient and Bland–Altman analysis

Measurement	Intrarater 1 ICC (95%CI)	Intrarater 2 ICC (95%CI)	Interrater ICC (95%CI)	95% CI of bias	Lower limit of agreement (95% CI)	Upper limit of agreement (95% CI)
Upper lip height (Sn-Sto)	0.88 (0.85–0.96)	0.93 (0.88–0.98)	0.85	− 0.26 to 0.14	− 1.37 (− 1.71 to − 1.02)	1.24 (0.90 to 1.58)
Lower lip height (Sl-Li)	0.85 (0.81–0.91)	0.89 (0.85–0.92)	0.82	− 0.36 to − 0.04	− 1.26 (− 1.53 to − 0.98)	0.86 (0.58 to 1.13)
Upper vermilion height (Ls-Sto)	0.94 (0.87–0.96)	0.97 (0.95–0.99)	0.92	− 0.01 to 0.16	− 0.46 (− 0.60 to − 0.32)	0.61 (0.47 to 0.75)
Lower vermilion height (Li-Sto)	0.98 (0.97–0.99)	0.99 (0.98–0.99)	0.98	− 0.09 to 0.01	− 0.37 (− 0.45 to − 0.28)	0.28 (0.20 to 0.37)
Philtral length (Sn-Ls)	0.99 (0.97–0.99)	0.99 (0.98–0.99)	0.98	− 0.04 to 0.09	− 0.38 (− 0.49 to − 0.28)	0.43 (0.33 to 0.54)
Philtral width (CphR-CphL)	0.89 (0.86–0.94)	0.94 (0.89–0.99)	0.84	− 0.14 to 0.10	− 0.81 (− 1.01 to − 0.60)	0.76 (0.56 to 0.97)
Nasolabial angle (Prn-Sn-Ls)	0.97 (0.94–0.99)	0.99 (0.98–0.99)	0.97	− 0.26 to 0.72	− 3.01 ( − 3.85 to − 2.16)	3.46 (2.62 to 4.31)
Labial fissure width (Lch-Rch)	0.99 (0.97–0.99)	0.99 (0.98–0.99)	0.98	− 0.10 to 0.11	− 0.69 ( − 0.87 to − 0.51)	0.70 (0.52 to 0.88)
Facial convexity (Gl-Sn-Pg’)	0.98 (0.95–0.99)	0.99 (0.98–0.99)	0.98	− 0.18 to 0.16	− 1.12 (− 1.41 to − 0.83)	1.10 (0.81 to 1.39)
Facial height (N’-Gn’)	0.95 (0.89–0.98)	0.98 (0.95–0.99)	0.93	− 0.31 to 0.40	− 2.33 (− 2.95 to − 1.71)	2.41 (1.80 to 3.03)
Nasal angle (N’-Prn-Sn)	0.99 (0.97–0.99)	0.99 (0.98–0.99)	0.96	0.01 to 0.68	− 1.87 (− 2.45 to − 1.29)	2.57 (1.99 to 3.14)
Nasofrontal angle (Gl-N’-Prn)	0.94 (0.90–0.96)	0.96 (0.92–0.98)	0.91	− 0.60 to 0.46	− 3.56 (− 4.47 to − 2.65)	3.42 (2.51 to 4.33)

(ICC: intraclass correlation coefficient; CI: confidence interval; Sn: Subnasale; Sto: Stomion; Sl: Sublabial; Prn: Pronasale; Ls: Labrale superius; Li: Labrale inferius; (L,R) ch: (Left, Right) cheilion; (L,R) Cph: (Left, Right) Crista Philtri; Gl: Glabella; N’: Nasion of soft tissue; Pg’: Pogonion of soft tissue; Gn’: Gnathion of soft tissue)

## Discussion

In this study, the FaceSCAN3D system was used to gather 3D facial information. Several portable and more affordable 3D scanners were also available for clinical usage. Currently, orthodontic clinical applications of 3D facial analysis include orthodontic diagnosis, treatment plan strategies, and pre-post treatment evaluation. For example, facial convexity largely influences the clinical decision of extraction treatment, and the increase of mentolabial angle is crucial for skeletal class II hyperdivergent patients after orthodontic treatment. However, due to the essence of orthodontic treatment, it takes about 2–3 years to finish a treatment, which limited the currently available 3D face models for relevant studies. Besides, with this method, 3D facial model will be more available for researchers, which to some extent, may eliminate the need for 3D facial scanning devices and relevant operation training. Thus, this study aimed to gain clinical acceptable 3D facial models for the previous finished orthodontic cases with only 2D records, which can offer significant data for future studies on 3D facial analysis.

In the field of computer science, computer vision (CV) techniques based on multi-ocular, binocular, or monocular views to reconstruct 3D models have matured. Depth information was lost during the projection of 3D items to 2D. Therefore, the task of 3D face modeling from 2D face photos is to essentially compensate for the lost depth information. However, current studies primarily focused on the robustness of the techniques, such as different light conditions and photo qualities, instead of modeling accuracy. With a steady photo-taking environment and camera setups, clinical 2D face photos gain the potential for high-quality 3D face modeling, which deserves further exploration. Currently, no clinical potential of these methods has been investigated.

A recently published systematic review concluded the current situation of 3D face model reconstruction based on 2D face photos [11]. The review noted that most studies focused on full faces modeling without evaluating accuracy adequately, and specific areas such as lips were rarely considered. Reconstruction of the perioral area, particularly the lip, is the most difficult [9]. Garrido et al. used 4 cameras focused on the lip and another 6 cameras for the full face area to reconstruct 3D models, and the mean error at the lip was approximately 3 mm [18]. Recently, a study used Character Creator to create different virtual characters to determine the gender judgments of body shape and motion [19]. Another study showed that the software is robust to complex light conditions [20].

As the results show, photorealistic 3D face models with accurate morphology and fine texture were constructed with the new method proposed in this study, and a good intrarater and interrater reliability of facial measurements were revealed with the method. However, during the experiment, approximately 30 min were required to reconstruct a facial model, which relied heavily on the user’s operating proficiency with the software. The most time-consuming process during the proposed workflow is the refinement of the front and side views of the models, which require detailed adjustment with various morph sliders. In this study, the RMSEs of both the entire facial area and perioral area between the test group and the gold standard remained within 2 mm, which indicated that the proposed method was clinically acceptable [16, 17]. To our knowledge, no published study so far has investigated the orthodontic clinical acceptance of accuracy for 3D facial model. Therefore, this study used 2 mm as the clinical acceptance of accuracy for 3D facial model based on the results of Kazandjian et al. [16]. Their study included orthodontists, oral and maxillofacial surgeons, and lay people as raters for 3D facial change evaluations of patients after orthognathic surgery, which we thought most suitable for reference for our study. As the color-coded maps show, large matching errors occurred primarily at the forehead, cheek, and nasal alar. These areas are irregularly curved surfaces with a feature-sparse nature, whose depth information could not be precisely determined with feature or counter of structures. During orthodontic or orthognathic treatment, forehead has always been chosen for facial registration before and after the treatment since it is relatively stable. Even though during 2D photo-taking, forehead is one of the largest exposed areas to the camera, the photo at front view lacks the information of depth of forehead, and the photo at lateral view can only provide the outline at lateral view, which is the mid-sagittal outline of forehead in most cases. The depth at temple or other area around forehead can only be gained by the texture or the shade information [21], which cause the inaccuracy of forehead reconstruction. Likewise, similar situation is for the cheek area. For nasal alars, the area with the maximum curvature changes throughout the facial area, and the depth information might be covered by the bulging cheek in the side view, should be carefully addressed. In future studies, 45° lateral view photos could be included to enhance the reconstruction accuracy in these areas. The primary deviations of the models came from the sagittal dimension (Y-axis) because it is difficult to determine the accurate depth of all feature points with the lateral view photos.

To validate the clinical implications of the reconstructed 3D face models, 12 commonly used linear or angular measurements were chosen based on a previous study [7, 15]. Despite the 5 measurements without significant differences (*P* > 0.05), the average deviations of the remaining 6 measurements, excluding facial height, showed no greater than 0.5 mm (linear measurements) or 1.2° (angular measurements), which is clinically acceptable. However, for the facial height, an average deviation of 1.5 mm was found between the gold standard and the 3D reconstructed face model. This significant difference (*P* < 0.05) may be due to the missing nasion area blocked by the protruding eyes in some lateral view photos. The differences between 3D facial models gained with different 3D imaging devices has been raised by researchers. Akan et al. [15] evaluated 3D facial models obtained by stereophotogrammetry and smartphone camera. Their results indicated significant changes of distance between inner commissures of right and left eye fissure and nasolabial angle, and RMS values were found between 0.58 and 1. Likewise, D’Ettorre et la [8]. carried out a similar study, and the 3D deviation results indicated an overlap percentage of 80% and 56% within the ranges of 1 mm and 0.5 mm discrepancy, respectively.

A preliminary study showed that the repositioning of 2D photos was critical for the accuracy of the final models. As Bas et al. reported, the focal lengths of different lenses and subjects yielded critical distortion effects for headshots, which could cause a maximum deviation of 2.5 mm for a face model [22]. In a pilot study, a testing board with grid lines was photographed under the same lighting environment, and the results suggested that a maximum distortion of 0.4 mm occurred at the corner of the photo. However, the accuracy of the reconstructed 3D faces with this new method showed that the distortion of the lens is tolerable.

The complex and time-consuming manual operation of this method is one of its primary limitations. Additionally, since Character Creator is a software developed for 3D animation, not for medical usage, no medically oriented features were included and thus can be developed for more clinical significance. However, this preliminary study aimed to validate the potential of this technical method via manual modeling. Based on the results of this study, an automatic, more accurate, and faster 3D face modeling method based on 2D photos is being developed. The results of this study also implied that less accurate areas, such as the forehead and cheeks, must be considered in more detail during algorithm development. Besides, as a primary study, this research aimed to investigate the possibility of gaining clinical acceptable 3D facial model based on conventional 2D clinical records. Subjects with obvious facial asymmetry or scars or other facial pathological changes were excluded. It is commonly believed that, within a certain number of views, more camera views provide more details of the object, which leads to higher accuracy of the 3D models.

## Conclusion

A 3D face modeling method based on 2D face photos was revealed and validated in this study. The reconstruction accuracy of this method is clinically acceptable for orthodontic measurement purposes, but the narrow clinical indication and labor-intensive operation remain problems.

## Data Availability

The datasets used and/or analyzed during the current study are available from the corresponding author on reasonable request.

## Abbreviations

3D: Three-dimensional
2D: Two-dimensional
CBCT: Cone-beam computed tomography
MRI: Magnetic resonance imaging
ABO: American Board of Orthodontics
Prn: Pronasale
Ls: Labrale superius
Li: Labrale inferius
Lch: Left cheilion
Rch: Right cheilion
Pg’: Pogonion of soft tissue
Gn’: Gnathion of soft tissue
Me’: Menton of soft tissue
CV: Computer vision
NR: Neural rendering
ML: Machine learning
